# Dangerous liaisons: Candidalysin and myeloid cells in candidiasis

**DOI:** 10.1371/journal.ppat.1014467

**Published:** 2026-07-31

**Authors:** Zi-Qi Gu, You-Yan Chen, Yu-Huan Tsai

**Affiliations:** 1 Laboratory of Host-Microbe Interactions and Cell Dynamics, Department of Medical Biotechnology and Laboratory Science, Chang Gung University, Taoyuan, Taiwan; 2 Institute of Microbiology and Immunology, College of Life Sciences, National Yang Ming Chiao Tung University, Taipei, Taiwan; 3 Division of General Surgery, Department of Surgery, Chang Gung Memorial Hospital, Taoyuan, Taiwan; 4 Center for Molecular and Clinical Immunology, Chang Gung University, Taoyuan, Taiwan; 5 Institute of Immunology and Translational Medicine, Chang Gung University, Taoyuan, Taiwan; University of Maryland, Baltimore, UNITED STATES OF AMERICA

*Candida albicans* causes a broad spectrum of diseases in humans, ranging from noninvasive superficial infections, such as chronic mucocutaneous candidiasis, to life-threatening systemic infections [1]. *C. albicans* produces candidalysin, a cytolytic peptide encoded by the *ECE1* (extent of cell elongation 1) gene [2]. Candidalysin is an amphipathic α-helical peptide containing two amyloidogenic regions. It is processed as the third peptide (Ece1-III) from the Ece1 protein by the proteases Kex2 and Kex1, which release the mature toxin into the extracellular environment [3]. Expressed primarily during the filamentous growth phase, candidalysin integrates into epithelial cell membranes, a process facilitated by interactions with sulfated glycosaminoglycans (GAGs), driving barrier disruption and orchestrated immune recruitment in mouse models of oropharyngeal and vulvovaginal candidiasis [3,4]. Candidalysin is also a potent host immune modulator. It triggers the secretion of pro-inflammatory cytokines and chemokines, most notably granulocyte-macrophage colony-stimulating factor (GM-CSF), which is essential for the migration and activation of myeloid cells [5,6]. In immunocompromised patients, such as those with *CARD9* deficiency, GM-CSF therapy has demonstrated significant potential in alleviating candidiasis [7,8]. Furthermore, the depletion of myeloid cells promotes fungal proliferation and increases host mortality, underscoring their critical role in antifungal defense [9]. While the foundational functions of candidalysin have been elegantly summarized previously [10], our understanding of its nuanced role in myeloid cell crosstalk continues to evolve. In this review, we synthesize current *in vitro* and *in vivo* findings on how this toxin coordinates complex interactions with myeloid cells, providing a framework to guide future research and therapeutic strategies in candidiasis.

## 1. Candidalysin induces host membrane damage to escape macrophagic control

**Fig 1:**
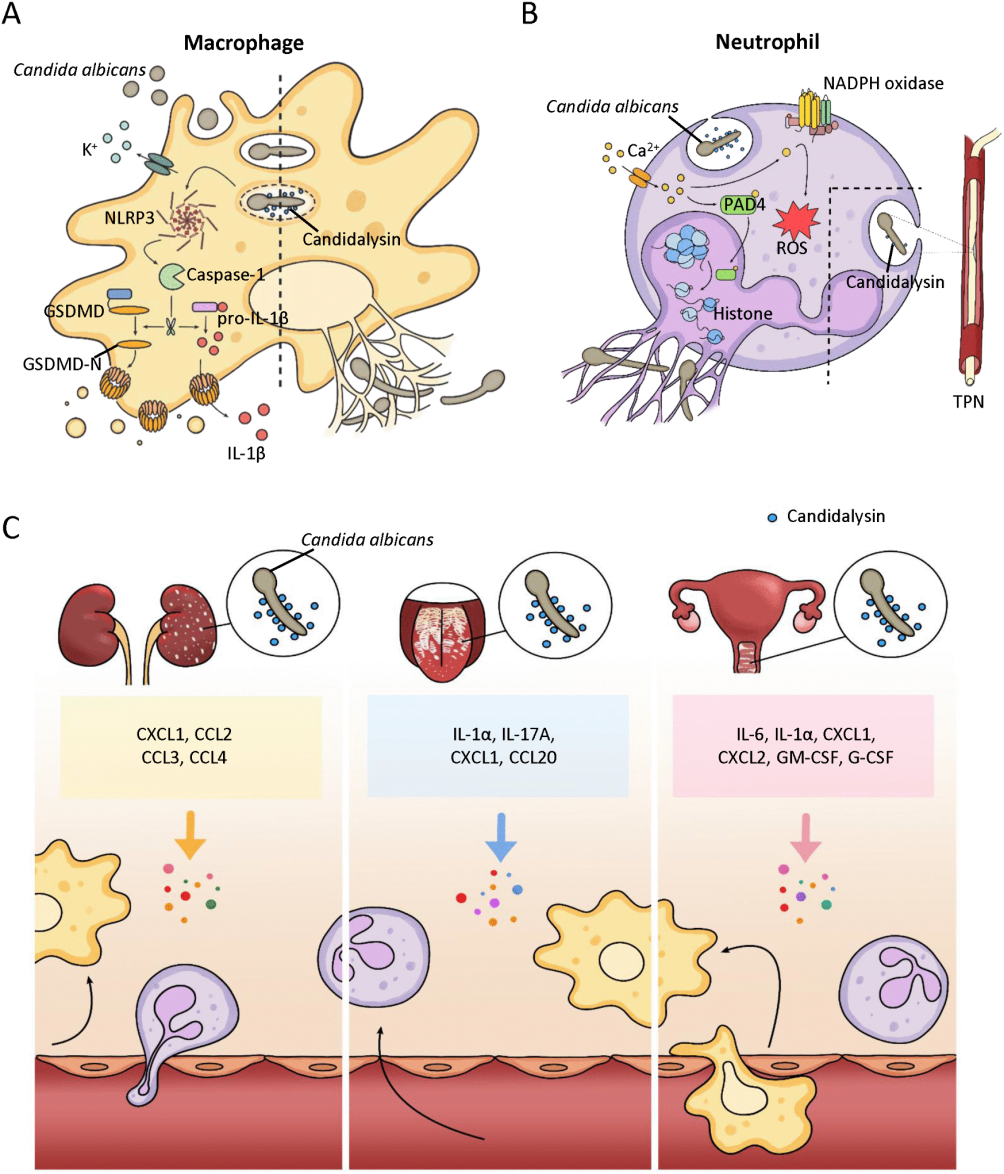
Candidalysin modulates myeloid cell functions and tissue immunopathology. **(A)** During macrophage phagocytosis of *C. albicans*, candidalysin triggers K⁺ efflux and activation of the NLRP3 inflammasome. Caspase-1-mediated cleavage of GSDMD and pro-IL-1β results in pore formation, IL-1β release, and pyroptotic cell death, thereby facilitating fungal escape from myeloid containment. In addition, candidalysin induces ETosis in myeloid cells. **(B)** Candidalysin activates neutrophils by inducing Ca²⁺ influx and NADPH oxidase-dependent reactive oxygen species production, together with PAD4-mediated histone citrullination. These signals drive chromatin decondensation and the release of NLS. In intravascular catheter environments, reduced candidalysin expression may enable *C. albicans* to evade NETosis. **(C)** Candidalysin induces different pro-inflammatory cytokine and chemokine profiles across tissues, resulting in divergent outcomes: reduced fungal load accompanied by pathogenic inflammation in the kidney, neutrophil recruitment associated with increased fungal load in the oral mucosa, and no impact on fungal burden in the vaginal mucosa.

Macrophages play a central role in clearing *C. albicans* from infected tissues [9]. Tissue resident macrophages phagocytose *C. albicans* to restrict its hyphal elongation. However, within the phagosome, *C. albicans* can undergo yeast-to-hyphae transition, generating mechanical forces that compromise phagosomal membrane integrity. In response, macrophages attempt to preserve phagosomal structure by triggering calcium-dependent endolysosomal fusion to aid in membrane repair [11]. Meanwhile, candidalysin expression by *C. albicans* or exogenous synthetic candidalysin can trigger potassium efflux and activation of the NLRP3 inflammasome to promote cleavage of gasdermin D (GSDMD), a pore-forming protein in the macrophage membrane, ultimately resulting in pyroptosis [12], and induces macrophage extracellular trap formation through ETosis, a distinct form of cell death [13]. Inhibition of GSDMD has been shown to reduce *C. albicans* escape and fungal burden in infected tissues. Beyond cell death, candidalysin-induced inflammasome activation drives the release of pro-inflammatory cytokines such as IL-1β, further shaping the immune landscape [6]. Thus, candidalysin facilitates *C. albicans* escape, simultaneously driving this dual program of cell death and inflammation within macrophages (Fig 1).

In contrast, *Candida auris*, another pathogenic but candidalysin-deficient species, kills macrophages via glycolysis-induced metabolic stress independently of NLRP3 activation [14]. This divergence may underscore the unique role of candidalysin in *C. albicans*-macrophage interactions.

## 2. Candidalysin activates neutrophil netotic program

Neutrophils are the most abundant circulating leukocytes and serve as a first line of defense against fungal infections [15]. Neutropenia or impaired neutrophil function predisposes individuals to systemic fungal infections [16]. Neutrophils combat *C. albicans* through multiple mechanisms, including phagocytosis, degranulation, and the release of neutrophil extracellular traps (NETs) [15]. NETs, composed of decondensed chromatin and antimicrobial proteins, trap and kill *C. albicans* hyphae, which are otherwise difficult to eliminate by phagocytosis alone [17,18], and were observed in intravascular catheters [19]. NET formation is critical for restricting *C. albicans* filamentous growth and occurs in intravascular catheters removed from candidemia patients [18,19].

Candidalysin triggers NET release by neutrophils under diverse conditions. However, synthetic candidalysin alone is insufficient to trigger classical NET formation. Instead, it induces the production of NET-like structures (NLS), characterized by more compact and less decondensed chromatin fibers than canonical NETs [18]. This NLS formation is partially dependent on NADPH oxidase-derived ROS, calcium influx, and activation of peptidylarginine deiminase 4 (PAD4), which leads to hypercitrullination of histones and primes chromatin for NETosis [18]. These findings suggest that candidalysin modulates neutrophil responses to *C. albicans* by influencing NET formation dynamics (Fig 1).

In a total parenteral nutrition (TPN) environment, human catheter-derived *C. albicans* isolates were found to express low levels of candidalysin, correlating with reduced NETosis and resistance to neutrophil-mediated biofilm clearance. Supplementation with synthetic candidalysin restored NETosis and enhanced neutrophil clearance of fungal biofilms. Notably, this NETosis-promoting effect of candidalysin was not observed under conventional cell culture conditions [19]. Indeed, TPN lipids have been shown to alter *C. albicans* transcriptome, particularly genes involved in filamentation [20]. These findings suggest that *C. albicans* clinical isolates may evolve to downregulate candidalysin expression as a strategy to evade neutrophil-mediated killing and persist in TPN-filled catheter environments. The impact of TPN components on candidalysin-mediated neutrophil responses warrants further investigation.

## 3. Context-dependent roles of candidalysin in tissue myeloid responses

Beyond direct interactions with circulating immune cells, *C. albicans* engages endothelial and epithelial cells, triggering candidalysin-dependent inflammatory responses that recruit myeloid cells [21–23]. In a gut colonization murine model, candidalysin expression promotes IL-6R-dependent systemic granulopoiesis and increases circulating neutrophil numbers, thereby enhancing long-lasting protection against systemic Gram-positive bacterial infections [24]. In oropharyngeal candidiasis, candidalysin induces expression of IL-1α, IL-17A, CCL20, and CXCL1 [22]. A significant portion of these known pathways depends on EGFR phosphorylation on epithelial cells, which requires matrix metalloproteinases, EGFR ligands, and calcium flux [21]. Downstream MAPK signaling (p38, ERK1/2) and AP-1/c-Fos activation amplify the release of IL-1α, IL-6, GM-CSF, and G-CSF [3]. Nevertheless, distinct EGFR-independent pathways operate concurrently and remain to be further explored [25]. Candidalysin also stimulates IL-17–producing CD4⁺ TCRαβ⁺ cells and drives oral megakaryocyte expansion, with platelets facilitating neutrophil influx [5,26]. Sustained candidalysin expression is required for robust inflammation, whereas transient expression in low-virulence strains enables epithelial penetration with minimal host response [27]. Thus, candidalysin acts in an expression-level-dependent manner, balancing successful colonization against the triggering of host inflammation that can restrict the microbes.

In vulvovaginal candidiasis, candidalysin induces neutrophil recruitment and elevation of IL-1α, IL-6, CXCL1, CXCL2, GM-CSF, and G-CSF [23]. However, in vaginal infections and oral infections under immunosuppressed conditions, these responses fail to control fungal load and instead cause pathology [22,23]. In the intravenous challenge model of *C. albicans* systemic infection, candidalysin promoted chemokine secretion (CXCL1, CCL2, CCL3, and CCL4), neutrophil recruitment, and reduced *C. albicans* fungal loads in the kidney [8]. Although fungal loads were decreased, the accompanying excessive inflammation drives high mortality [8]. In summary, candidalysin consistently recruits neutrophils across tissues, but its outcomes range from ineffective control to lethal immunopathology, depending on the infection site (Fig 1). This divergence may be driven by distinct neutrophil phenotypes or functional heterogeneity across different tissue microenvironments, which warrant further investigation.

## Conclusions and future directions

With the standard clinical reference strain SC5314, candidalysin has been demonstrated to act as a master molecular modulator governing the delicate equilibrium between fungal persistence and host immunopathology. While essential for alerting host defenses via macrophage inflammasome activation and formation of NETs, high virulence expression of this strain often shifts this balance toward self-destructive tissue pathology. Crucially, emerging evidence indicates that candidalysin is vital in sustaining stable fungal oral commensalism by a low-virulent human isolate, rather than driving mucosal destruction by the high-virulent SC5314, underscoring a strain-dependent manner of this dual nature [27]. Similarly, in the TPN environment, some human catheter isolates exhibit low candidalysin expression to evade neutrophil attack, while SC5314 induces inflammation dependent on candidalysin [19]. While current *in vivo* murine models of lethal immunopathology rely heavily on SC5314 and its derivatives, this widespread experimental dependence risks biasing our understanding of candidalysin function and virulence across the broader landscape of clinical isolates.

To transition these insights into viable clinical applications, several critical frontiers must be explored. First, it is essential to investigate the exact molecular networks by which microenvironmental cues, such as host nutritional lipids, modulate candidalysin expression to promote persistent colonization. Crucially, while *in vitro* studies demonstrate that candidalysin is essential for intestinal epithelial damage and subsequent fungal translocation [28], *in vivo* oral-gastric dissemination by even the high-virulent SC5314 strain necessitates an immunocompromised host [29]. This discrepancy underscores that *in vivo*, *C. albicans* encounters a complex, multicellular microenvironment containing highly specialized cell types, such as colonic macrophages that extend balloon-like protrusions to limit candidalysin absorption and preserve epithelial integrity [30]. Parallel to these cellular complexities, future studies must evaluate whether synthetic candidalysin accurately recapitulates the precise spatial gradients of native toxin delivered by live *C. albicans*. Resolving these discrepancies between *in vitro* and *in vivo* models will clarify whether candidalysin modulates myeloid cells directly or acts indirectly via epithelia-derived signals. Furthermore, the field must determine how candidalysin-induced signaling shapes localized tissue microenvironments and primes distinct neutrophil phenotypes, explaining why identical immune responses yield protective clearance in certain organs but severe pathology in others. Alongside these heavily investigated phagocytes, expanding our focus to elucidate how candidalysin interacts with other essential myeloid populations, such as monocytes, dendritic cells, and distinct mononuclear phagocyte subsets, remains a crucial gap in the current literature.

From a translational standpoint, while directly neutralizing candidalysin offers a viable avenue to prevent epithelial damage by SC5314 [31], this intervention can potentially compromise host immune surveillance and drive *C. albicans* immune evasion. To safely navigate this challenge, future clinical frameworks must adapt therapeutic strategies based on strain profiles, for instance, infections involving low-virulence expressing variants should focus on disrupting biofilm persistence rather than neutralizing acute cytotoxicity. Alternatively, precisely targeting downstream host effectors, such as GSDMD, may offer a targeted strategy to decouple toxin-mediated tissue destruction from essential antifungal immunity. Taken together, unraveling these localized, strain-specific dynamics will be key to developing niche-optimized immunomodulatory strategies.
